# Lipidomic Profiling of Colonic Contents in Mice with Acute *Toxoplasma gondii* Infection

**DOI:** 10.3390/microorganisms14040824

**Published:** 2026-04-03

**Authors:** Cai-Qin Deng, Wen-Jie Cheng, Yuan-Lan Wang, Yi-Dan Wang, Feng-Cai Zou, Xing-Quan Zhu, Zhao Li

**Affiliations:** 1 Animal Research and Resource Center, School of Life Sciences, Yunnan University, Kunming 650500, China; dengcaiqin1998@163.com (C.-Q.D.); 15211045113@163.com (Y.-L.W.); 2 The Yunnan Key Laboratory of Veterinary Etiological Biology, College of Veterinary Medicine, Yunnan Agricultural University, Kunming 650201, China; wangyidan318@163.com (Y.-D.W.); zfc1207@vip.163.com (F.-C.Z.); xingquanzhu1@hotmail.com (X.-Q.Z.); 3 Faculty of Animal Science and Technology, Yunnan Agricultural University, Kunming 650201, China; cwj210365@163.com; 4 Shanxi Key Laboratory of Animal Disease Research, College of Veterinary Medicine, Shanxi Agricultural University, Jinzhong 030801, China

**Keywords:** *Toxoplasma gondii*, acute infection, lipidomics, colonic contents

## Abstract

Background: While *Toxoplasma gondii* infection poses a significant health threat, its impact on the localized intestinal lipid metabolism remains unclear. Methods: Thus, this study established an acute infection model in BALB/cJ mice and analyzed the colonic contents collected 10 days post-infection via untargeted lipidomics. The lipid profiles of the two groups diverged substantially, with a clear separation evident between infection and control conditions. Results: We identified 65 upregulated and 87 downregulated differential lipids, primarily falling into the glycerophospholipids and sphingolipids categories. Pathway enrichment analysis revealed that the choline metabolism in cancer and glycerophospholipid metabolism pathways was pinpointed as being among the most perturbed following infection. Correlation and network analyses further suggested that key molecules, such as LPC (20:4) and LPA (18:0), may mediate these metabolic pathway abnormalities by regulating related enzymatic activities. Conclusions: This systematic characterization of the intestinal lipid metabolic landscape in mice during acute *T. gondii* infection revealed the host intestinal lipid metabolic reprogramming induced by *T. gondii* infection. The findings offer a novel metabolic perspective for understanding *T. gondii* pathogenesis and host–parasite interactions.

## 1. Introduction

As an obligate intracellular parasite, the protozoan *Toxoplasma gondii* is capable of infecting numerous warm-blooded animals and humans [1]. The global human infection rate with *T. gondii* is approximately 30% [2], posing a severe health threat. Humans are primarily infected orally by ingesting meat contaminated with *T. gondii* tissue cysts or consuming food contaminated with sporulated oocysts [3]. While *T. gondii* infection in immunocompetent individuals is mostly asymptomatic, acute infection in immunocompromised individuals, pregnant women, and fetuses can have severe consequences, such as encephalitis, miscarriage, and congenital malformations [4,5]. The pathogenic mechanisms of *T. gondii* are complex, involving parasite-host cell interactions, immune evasion, and metabolic reprogramming [2,6,7]. Understanding the physiological alterations induced by *T. gondii* in mammalian hosts is crucial, considering their profound clinical implications.

The intestine is the primary portal of entry for *T. gondii* and a critical site for complex metabolic and immune interactions between the parasite and the host [8]. By significantly altering the microbiota structure, mucosal barrier, and metabolite composition in the intestinal microenvironment, parasite infection may directly affect disease progression and host responses [9]. Resulting from host digestion, microbial metabolism, and parasite activity, the metabolite composition of the intestinal contents dynamically reflects the physiological and pathological states during infection [10,11]. Lipids are essential constituents of cell membranes, energy storage molecules, and signaling mediators, thereby playing complex roles in host–parasite interactions. For one thing, *T. gondii* is a strict lipid auxotroph that relies on host lipid biosynthesis pathways to provide key components (e.g., cholesterol and fatty acids) for its infectious progeny and to survive within the host [12]. For another, various lipid metabolites (e.g., sphingolipids, phospholipids, and endocannabinoids) directly modulate host immune and inflammatory responses [13]. Therefore, elucidating the dynamic reprogramming of intestinal lipid metabolism during acute *T. gondii* infection sheds critical light on the mechanisms of host–parasite interactions.

Previous studies predominantly focused on gene regulation, immune responses, and histopathological changes induced by *T. gondii* infection, while the dynamic intestinal metabolism landscape, particularly from the lipidomics perspective, received limited attention [4,14]. Lipidomics has emerged as a crucial discipline under the umbrella of metabolomics, encompassing the systematic identification and quantification of diverse lipid species and their molecular structures in biological samples. It has emerged as a valuable tool for identifying abnormal metabolic pathways, biomarkers, and associated mechanisms [15,16]. Rapid advances in mass spectrometry over the past years have enabled high-throughput, high-sensitivity lipid detection, offering powerful tools for infectious disease research from the metabolic perspective [17]. Lipidomics can help identify infection-specific lipid profiles and decipher the functional roles of lipid metabolic networks in host defense or parasite pathogenesis [18], thereby providing new insights into the intestinal pathogenic mechanisms of *T. gondii*.

Using an acute *T. gondii* infection mouse model, this study systematically characterizes the intestinal lipid metabolic landscape in the early infection phase. Colonic contents collected 10 days post-infection are subjected to untargeted lipidomics to identify key differential lipids and affected metabolic pathways. These findings advance our understanding of *T. gondii*’s intestinal pathogenesis and associated metabolic crosstalk by elucidating novel mechanisms at the host–parasite interface.

## 2. Materials and Methods

### 2.1. Ethics Statement

Prior to commencement, the study was formally authorized by the Institutional Animal Care and Use Committee of Yunnan University (Approval No. 20241024). All procedures subsequently adhered to the national guidelines for the care and use of laboratory animals in China.

### 2.2. Chemicals and Reagents

Liquid chromatography–mass spectrometry (LC-MS) grade methanol (A454-4) and acetonitrile (A998-4) were obtained from Thermo Fisher Scientific (Waltham, MA, USA). Ammonium formate (Honeywell Fluka, Morris Plains, NJ, USA; Cat# 17843-250 G) and formic acid (DIMKA, Philadelphia, PA, USA; Cat# 50144-50 mL) were sourced from their respective commercial suppliers.

### 2.3. Animal Experiments

Specific pathogen-free (SPF) female ICR and BALB/cJ mice (6–8 weeks old) were sourced from the Laboratory Animal Center of Yunnan University. Upon arrival, all animals were maintained under standard SPF conditions with controlled temperature (22 ± 2 °C), humidity (50–60%), and a 12 h light/dark cycle, with ad libitum access to food and water. Tachyzoites of *T. gondii* type II ME49 strain were cryopreserved in liquid nitrogen. After thawing, they were used to infect HFF cells. At 12 h post-infection, non-invaded tachyzoites were washed away with cell culture medium. At 48–60 h post-infection, tachyzoites were collected, purified, and counted. Each ICR mouse was then intraperitoneally injected with 500 tachyzoites of these freshly collected tachyzoites. Following euthanasia at 30 days post-infection, brain tissues from ICR mice were harvested and homogenized. The tissue cysts obtained were subsequently used to infect female BALB/cJ mice. Female BALB/cJ mice (*n* = 12) were randomly divided into the infected group (BCA [Brain Cyst Administration] group, *n* = 6) that received oral gavage of 100 μL of phosphate-buffered solution (PBS) suspension containing 50 brain cysts per mouse and the control group (BCN [Brain Cyst Negative] group, *n* = 6) that received an equal volume (100 μL) of PBS by oral gavage.

### 2.4. Sample Collection

On day 10 post-infection (acute infection phase), BALB/cJ mice were euthanized by carbon dioxide asphyxiation. Following collection, the mouse colonic contents were immediately flash-frozen in liquid nitrogen and subsequently processed for lipidomic analysis.

### 2.5. Metabolite Extraction

Each colonic content sample (25 mg) was transferred into a 2 mL centrifuge tube. Then, 800 µL of pre-cooled dichloromethane/methanol (3:1, *v*/*v*) was added, followed by bead grinding for 5 min. The mixture was treated with ice-bath sonication for 10 min and subsequently maintained at −20 °C overnight. After centrifugation at 25,000× *g* for 15 min at 4 °C, 600 µL of the supernatant was collected and dried in a freeze concentrator. The extract was reconstituted in 600 µL of lipid resuspension solution (with an isopropanol to acetonitrile to water ratio of 2:1:1), vortexed for 10 min, and then sonicated in an ice bath for 10 min. The mixture was centrifuged again at 25,000× *g* for 15 min at 4 °C. The final supernatant was subjected to UPLC-MS analysis; concurrently, quality control (QC) samples were prepared by combining a 20 µL aliquot from each experimental sample.

### 2.6. UPLC-MS Analysis

Lipidomic analysis was performed using a UPLC system coupled to a Q-Exactive mass spectrometer (Thermo Fisher Scientific, USA). Chromatographic separation was carried out on a CSH C18 column (1.7 µm, 2.1 mm × 100 mm, Waters, Milford, MA, USA) at 55 °C, with a flow rate of 0.4 mL/min and an injection volume of 5 µL. The mobile phases for positive ion mode were (A) 60% acetonitrile/water containing 10 mM ammonium formate and 0.1% formic acid, and (B) 90% isopropanol/10% acetonitrile with the same additives. For negative ion mode, the additives were 10 mM ammonium formate in both phases. The elution gradient was as follows: 0–2 min, 40–43% B; 2–2.1 min, 43–50% B; 2.1–7 min, 50–54% B; 7–7.1 min, 54–70% B; 7.1–13 min, 70–99% B; 13–13.1 min, 99–40% B; 13.1–15 min, 40% B. Full-scan MS and MS/MS data were acquired and subsequently processed using LipidSearch v.4.1 (Thermo Fisher Scientific, USA) for lipid identification and relative quantification.

### 2.7. Data Analysis

All experimental data are presented as mean ± standard deviation (SD). Differences between group means were analyzed using SPSS 20.0 (IBM, Armonk, NY, USA). Graphs were prepared using GraphPad Prism 8. Differences with *p* < 0.05 were considered statistically significant.

## 3. Results

### 3.1. Information from Lipidomics Data

**Figure 1:**
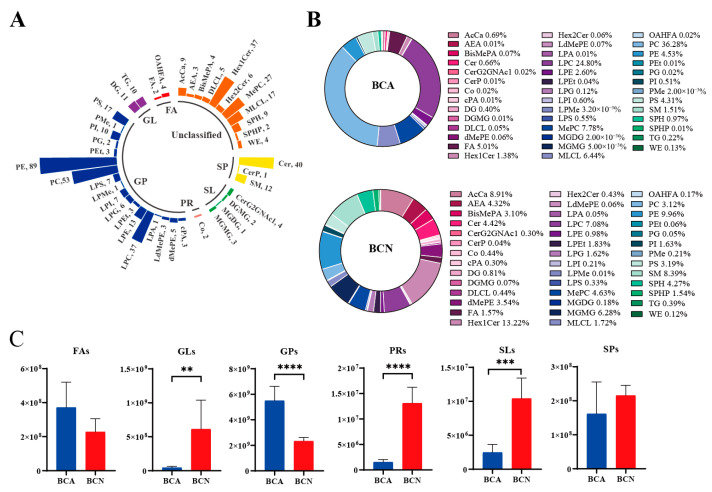
Lipid profile analysis of colonic contents in mice with acute *T. gondii* infection. (**A**) Distribution of lipids identified in colonic contents from the BCA and BCN groups. Different colors are employed in the bar graph to visually distinguish between lipid classes. Abbreviations in parentheses represent lipid subclasses, and the numbers indicate the counts of differential lipid species identified within each subclass. (**B**) Donut graph showing the percentage composition of lipid subclasses in the BCA and BCN groups. (**C**) Comparison of the relative levels of six major lipid categories between the BCA and BCN groups. ** *p* < 0.01, *** *p* < 0.001, **** *p* < 0.0001.

The dynamic changes in lipids following acute *T. gondii* infection were investigated by characterizing the lipid profiles in the *T. gondii*-infected BCA group and the non-infected control BCN group using UPLC-MS. This approach led to the identification of 476 lipids across 41 subclasses (Supplementary Table S1). The majority of the lipids were phosphatidylethanolamines (PE; 89), phosphatidylcholine (PC; 53), ceramide (Cer; 40), lysophosphatidic acids (LPC; 37), hexosylceramide (Hex1Cer; 37), and methylated phosphatidylcholine (MePC; 27). All lipids were further divided into glycerophospholipids (GPs; 261), sphingolipids (SPs; 53), glycerolipids (GLs; 21), saccharolipids (SLs; 10), fatty acids (Fas; 6), and prenol lipids (PRs; 2), with an additional 123 species remaining unclassified (Figure 1A). The BCA and BCN groups showed similar subclass composition, but their proportions of each lipid subclass varied significantly (Figure 1B). Compared to the BCN group, the BCA group showed decreased proportions of PE (9.96% vs. 4.53%), Cer (4.42% vs. 0.66%), SM (8.39% vs. 1.51%), and Hex1Cer (13.22% vs. 1.38%), but increased proportions of PC (3.12% vs. 36.28%), LPC (7.08% vs. 24.80%), and LPE (0.98% vs. 2.60%). *T. gondii* infection altered the levels of the six major lipid categories. The BCA group displayed a significant enrichment of GPs (*p* < 0.0001), whereas the BCN group showed elevated levels of GLs, PRs, and SLs (*p* < 0.01) (Figure 1C).

### 3.2. Multivariate Data Analytics

**Figure 2:**
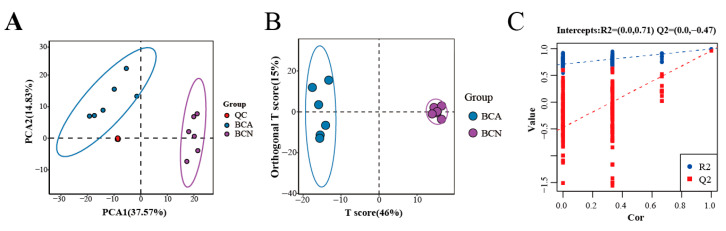
Multivariate data analytics of lipidomic profiles in mouse colonic contents. (**A**) PCA score plot of QC samples. (**B**) OPLS-DA score plot of the colonic contents lipid profiles in the BCA and BCN groups. (**C**) Permutation test of the colonic contents lipid profiles.

Principal component analysis (PCA) revealed a distinct separation between the BCA and BCN groups. The QC samples were tightly clustered, demonstrating the stability and reliability of the analytical system (Figure 2A). Orthogonal partial least squares-discriminant analysis (OPLS-DA) demonstrated a clear and complete separation between the two groups (Figure 2B). The robustness of the OPLS-DA model was assessed by response permutation testing. The R2Y (0.994) and Q2 (0.962) values confirmed the model’s strong predictive ability and reliability without overfitting (Figure 2C). Acute *T. gondii* infection can induce lipid metabolism disorders in the mouse intestines.

### 3.3. Identification of Lipid Biomarkers and Pathways

**Figure 3:**
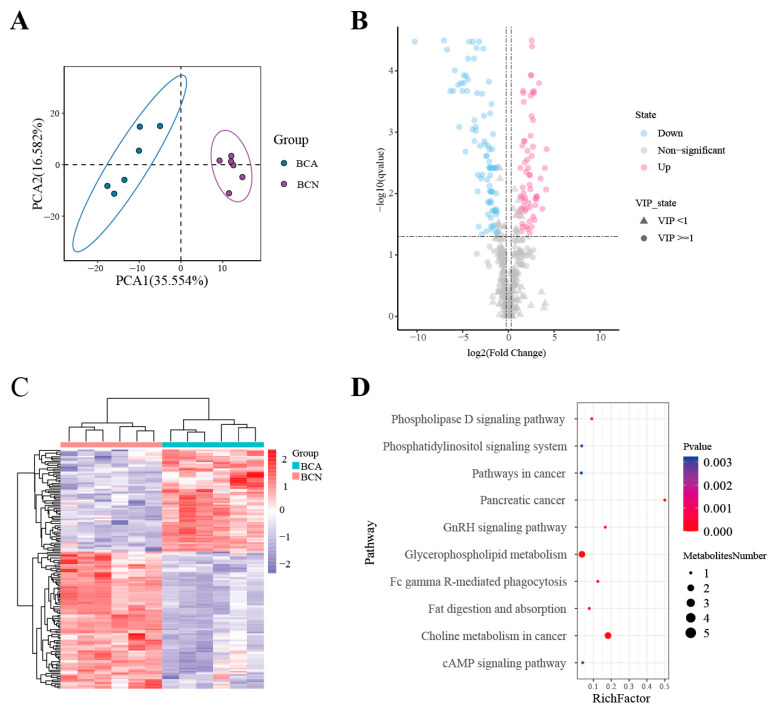
Screening of differential lipids and pathway analysis. (**A**) PCA score plot. (**B**) Volcanic plot of differential lipids. (**C**) Heatmap of lipid classes. (**D**) Enriched KEGG pathways.

The PCA revealed a clear segregation of the BCA and BCN groups into separate branches, reflecting significantly distinct lipid metabolic profiles in their colonic contents (Figure 3A). Potential lipid biomarkers were selected by applying multiple filtering criteria: a variable importance in projection (VIP) > 1, a fold change (FC) beyond the range of 0.83 to 1.2, and a q-value < 0.05. Comparative analysis revealed 152 differentially lipid species across the BCA and BCN groups. Relative to the BCN group, the BCA group exhibited 65 up-regulated and 87 down-regulated lipid species (Figure 3B). A heatmap was plotted to visualize the alterations in lipid classes of the mouse colonic contents (Figure 3C) (Supplementary Table S1). Pathway enrichment analysis was performed to identify changes in key lipid metabolism-related pathways. Acute *T. gondii* infection significantly affected several pathways, including Choline metabolism in cancer and Glycerophospholipid metabolism, Fc gamma R-mediated phagocytosis pathway, and GnRH signaling pathway. Among these, Glycerophospholipid metabolism and Choline metabolism in cancer may play crucial roles in the progression of acute *T. gondii* infection (Figure 3D).

### 3.4. Integrated Correlation and Network Analysis of Lipid Metabolism

**Figure 4:**
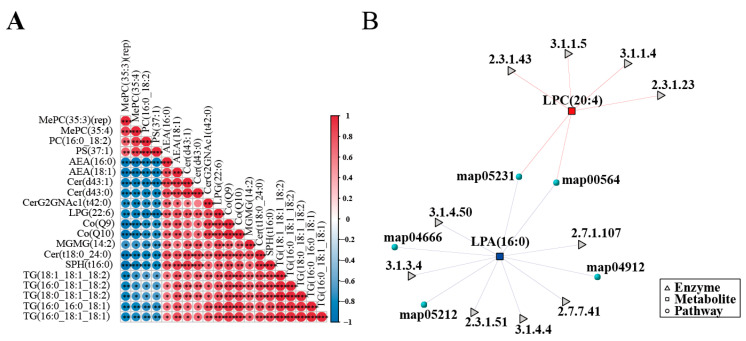
Correlation and network analysis of altered lipid metabolism. (**A**) Spearman correlation analysis of differential lipid metabolites. (**B**) Metabolite-enzyme-pathway interaction network. * *p* < 0.05, ** *p* < 0.01, and *** *p* < 0.001.

The overall changes and interactions within the mouse intestinal lipid metabolism network following *T. gondii* infection were further investigated via Spearman correlation analysis using the top 20 differential metabolites ranked by the lowest q-values. The results revealed significant positive correlations among triglyceride (TG) molecules, including TG (16:0_16:0_18:1), TG (16:0_18:1_18:1), TG (16:0_18:1_18:2), TG (18:0_18:1_18:2), and TG (18:1_18:1_18:2), with correlation coefficients > 0.78. In addition, coenzyme Q9 (CoQ9) and coenzyme Q10 (CoQ10) also showed significant positive correlations with TG lipids. In contrast, phospholipids such as PC, PS, and MePC exhibited significant negative correlations with the endocannabinoid AEA and ceramide (Cer) metabolites (Figure 4A). The metabolite-enzyme-pathway interaction network further elucidated the metabolic regulatory mechanisms of two key differential metabolites: lysophosphatidylcholine LPC (20:4) and lysophosphatidic acid LPA (18:0). LPC (20:4) is primarily catalyzed by enzymes such as EC 2.3.1.43 and EC 3.1.1.5, involved in processes like the phospholipase D signaling pathway, thereby linking to glycerophospholipid metabolism. Meanwhile, LPA (18:0) is metabolized by enzymes including EC 3.1.1.50 and EC 2.7.1.41, thereby entering pathways like choline metabolism in cancer. These interactions suggest that the disruption of the glycerophospholipid metabolism in the intestine following *T. gondii* infection may be mediated by the dysregulation of these key metabolites and their associated enzymes (Figure 4B).

## 4. Discussion

Despite prior metabolomics studies that have characterized metabolic alterations induced by *T. gondii* (strain 14) infection across multiple murine tissues, such as the liver, spleen, and cerebellum, research on lipidomic changes in mouse intestinal contents during infection remains relatively scarce [14,19,20]. For the first time, this study used non-targeted lipidomics to systematically characterize the lipid metabolic landscape of mouse colonic contents during the acute phase (day 10 post-infection) of *T. gondii* infection. The results demonstrated that acute *T. gondii* infection significantly disrupts the local intestinal lipid metabolic homeostasis, consistent with previous findings that *T. gondii* infection reprograms host metabolism [21]. As an obligate intracellular parasite, *T. gondii* hijacks host lipid resources for its own proliferation and interferes with host physiological functions. The significant dysregulation of 152 lipids after infection indicates severe disruption to the host lipid metabolism, aligning with earlier conclusions [22].

The observed alterations in various subclasses of glycerophospholipids (GPs) confirmed the central role of these structural phospholipids in host–parasite interactions. Phosphatidylcholine (PC) and phosphatidylethanolamine (PE) are fundamental structural constituents of all biological membranes, including the plasma and organelle membranes [23]. Due to its obligate intracellular parasitic nature, *T. gondii* relies on rapid membrane synthesis and remodeling for host–cell invasion, intracellular replication, and progeny tachyzoite release [24]. *T. gondii* has developed a sophisticated glycerophospholipid metabolic network to synthesize key precursors through multiple complementary pathways and directly acquire them from the host cell [25]. Therefore, *T. gondii* depends heavily on host-supplied phospholipid precursors. The significant enrichment of pathways like choline metabolism in cancer and glycerophospholipid metabolism, as observed in this study, likely reflects compensatory metabolic reprogramming in various host cells, such as intestinal epithelial cells and immune cells, in response to this substantial demand [8]. Previous research has shown that *T. gondii* can modulate the transcriptional and metabolic networks of host cells, upregulating the expression of lipid synthesis-related genes to meet its own needs [22,26]. The altered abundance of molecules such as PC, LPC, PE, and LPE detected in this study likely mirrors this dynamic process. Among them, the lysophospholipids (LPC, LPE) are both products of membrane phospholipid degradation and important signaling molecules involved in regulating inflammatory responses and immune-cell migration [27]. Their changes post-infection suggest that lipid remodeling may supply metabolites while modulating signaling [15]. Besides competing for lipid resources, *T. gondii* infection also activates ATF4 through the HRI/OMA1-mediated integrated stress response. This in turn enhances mitochondrial one-carbon metabolism to sequester folate, thereby ultimately restricting parasite deoxythymidylate synthesis and proliferation. These findings further underscore the core strategy by which the host defends against infection through multifaceted metabolic reprogramming [28].

In addition to glycerophospholipids, significant alterations were observed in other lipid classes, including sphingolipids (Cer, SM, Hex1Cer) and glycerolipids (GLs). Ceramide (Cer) is central to sphingolipid metabolism, and its accumulation can induce oxidative stress, making it a key mediator of apoptosis [29]. *T. gondii* infection can cause intestinal epithelial damage [9], a process that may be linked to infection-induced Cer accumulation, which disrupts tight-junction components such as occludin and claudin-4 and increases intestinal permeability [30]. Abnormal Cer expression is also possibly involved in regulating intestinal inflammatory responses. Meanwhile, sphingolipids and cholesterol form “lipid raft” microdomains in cell membranes, which are crucial for receptor clustering and signal transduction during *T. gondii* invasion [31]. Studies showed that Hex1Cer is significantly positively correlated with changes in Tnf-α, Cxcl1, Cxcl2, Cxcl5, Ccl2, Ccl12, and IL-1β [32]. Hexosylceramide (Hex1Cer) is a member of the sphingolipid family and participates in cell recognition and adhesion, and its changes may affect immune factors and, in turn, the progression of inflammatory bowel disease [33]. Glycerolipids (primarily triglycerides) serve as primary energy reservoirs, and their specific subclasses (mono- and di-glycerides) have demonstrated direct antimicrobial activity and immunomodulatory functions [34,35]. The observed glycerolipid composition alterations in the host may indicate a functional shift of these lipids from an energy reservoir to immune effectors and signaling molecules, which affects the host’s immune defense response. The coordinated changes across multiple lipid classes collectively indicate that *T. gondii* infection triggers a systemic intestinal lipid reprogramming in the host.

Correlation analysis revealed that TG family members formed a strong positive correlation cluster and were also significantly positively correlated with coenzyme Q (CoQ9 and CoQ10). CoQ10 may act as an endogenous ligand, transmitting information about mitochondrial, peroxisomal, and cellular redox states to PPAR receptors. PPAR activation upregulates the expression of genes involved in fatty acid oxidation, promoting the entry of fatty acids derived from the hydrolysis of stored TGs into the β-oxidation pathway for energy production [36]. Meanwhile, CoQ supports this process by participating in the electron transport chain, forming a synergistic feedback regulatory mechanism. Phospholipids (PC, PS) showed a negative correlation with the endocannabinoid AEA. Notably, intestinal AEA levels were elevated and involved in immune regulation [37]. Infected cells may need to dynamically balance the demands of maintaining membrane structural stability and activating specific signaling pathways.

KEGG enrichment analysis indicated that the most significantly altered pathways after *T. gondii* infection were glycerophospholipid and choline metabolism in cancer. Notably, “choline metabolism in cancer” is a standard KEGG pathway name reflecting the enrichment of choline-related lipid metabolites, and its identification here does not imply oncogenic transformation but rather highlights the central role of choline metabolism in host cellular processes during infection. These two pathways are highly interconnected and jointly point to active membrane lipid synthesis and remodeling during infection. As primary components of cell membranes, changes in glycerophospholipid content alter the morphology of intestinal epithelial cell membranes, and the affected membrane integrity and permeability may facilitate *T. gondii* invasion and dissemination [38]. The enrichment of the choline metabolism in cancer pathway points to a mechanism whereby *T. gondii* infection may subvert the host’s homeostatic choline regulatory program. Previous research showed upregulated expression of the choline transporter (CTL1), increased choline uptake, and improved PC synthesis in LPS-activated macrophages, which promoted pro-inflammatory cytokine release [39]. In an ovarian cancer model, dysregulated choline metabolism was found to affect cell proliferation by modulating the AKT signaling pathway [40]. Such evidence indicates that the choline metabolism is a key regulatory node that simultaneously influences inflammatory responses and cell proliferation signals. Thus, targeting the choline/phospholipid metabolism may prove a novel strategy for anti-parasitic drug development [41]. Several compounds targeting choline/phospholipid metabolism have shown promising anti-*Toxoplasma gondii* activity. Choline analogs, such as the JCP series (JCP341, JCP342, JCP343, JCP348, and JCP383), inhibit the CDP-choline pathway by interfering with choline kinase and choline/ethanolamine phosphotransferase, thereby blocking phosphatidylcholine synthesis in apicomplexan parasites [25]. Additionally, the natural product catechin gallate (CG) has been reported to induce metabolomic and lipidomic alterations in *T. gondii* tachyzoites, leading to reduced phosphocholine production and impaired parasite proliferation [18]. These findings highlight the potential of targeting the choline metabolic axis for the development of novel antitoxoplasmal agents.

It has been shown that during *T. gondii* infection (days 7, 14, 21), the glycerophospholipid and choline metabolism pathways in cancer remain persistently affected in the mouse cerebellum [20]. This study found that these same pathways were significantly perturbed in colonic contents at a comparable infection phase. This cross-tissue metabolic commonality points to a systemic host disturbance involving multiple core lipid metabolic networks under *T. gondii* infection. These findings open new research directions for understanding the system-level regulation of host–parasite interactions during *T. gondii* infection. The metabolite-enzyme-pathway interaction network revealed LPC (20:4) and LPA (18:0) as key node molecules that mediate pathway abnormalities by regulating phospholipase-related enzymatic reactions, further confirming that *T. gondii* can precisely modulate host lipid metabolism by targeting core metabolic enzymes [12].

In this study, colonic contents were collected from euthanized mice at a single time point (day 10 post-infection) rather than through longitudinal fecal sampling from live animals. Although longitudinal fecal collection enables dynamic tracking and reduces inter-individual variability, it has notable limitations. Fecal samples represent a mixture of the entire intestinal content, making it difficult to pinpoint regional changes in the colon. Moreover, variations in fecal transit and excretion time among individuals may introduce variability due to lipid degradation or microbial metabolism. In contrast, collection after euthanasia ensures precise anatomical localization, consistent sampling time, and immediate cryopreservation, thereby maximizing the fidelity of the lipidomic profile. This approach is suitable for a cross-sectional study aimed at systematically characterizing the static lipid landscape during the acute phase of infection. Therefore, although the present design does not capture temporal dynamics, it provides a solid foundation for subsequent time-course studies and mechanistic investigations.

Although this study systematically depicted the lipidomic landscape of colonic contents during acute *T. gondii* infection, several limitations remain. First, this study focused solely on metabolic changes at day 10 post-infection and, therefore, cannot reflect the dynamic evolution of lipid metabolism. Second, the lipids in colonic contents originate from complex sources, including sloughed host intestinal epithelial cells, secreted bile, and microbial metabolites, which require further distinction. Finally, the functional impact of the differential lipids requires further validation through in vitro and in vivo experiments. Future studies should construct dynamic lipid metabolic profiles at various time points following infection to clarify the temporal characteristics of metabolic remodeling. Then, the lipid changes identified herein can be integrated with data on gut microbiota composition, host transcriptomics, and proteomics, and such a “multi-omics” analysis can build a more comprehensive “infection-metabolism-immune” interaction network [42,43]. Gene-knockout mice or metabolic enzyme inhibitors can be utilized for functional validation to elucidate the specific roles of particular lipids during *T. gondii* infection.

## 5. Conclusions

Through lipidomic analysis, this study revealed that acute *T. gondii* infection significantly reshapes the mouse intestinal lipid metabolic profile. Lipidomic profiling revealed 152 significantly dysregulated lipids, with glycerophospholipid and choline metabolism emerging as the central altered pathways. Key molecules such as LPC (20:4) and LPA (18:0) mediated pathway abnormalities by regulating enzymatic reactions. These findings added a crucial metabolic dimension to the knowledge of *T. gondii* pathogenesis while laying a theoretical foundation for identifying potential diagnostic biomarkers and developing novel adjunctive therapeutic strategies based on metabolic intervention.

## Data Availability

The original contributions presented in this study are included in the article/Supplementary Material. Further inquiries can be directed to the corresponding author.

## Supplementary Materials

The following supporting information can be downloaded at: https://www.mdpi.com/article/10.3390/microorganisms14040824/s1, Table S1. The datasets supporting the findings of this article are included within the paper.
